# Addressing the Global Burden of Cardiovascular Diseases; Need for Scalable and Sustainable Frameworks

**DOI:** 10.5334/gh.1139

**Published:** 2022-07-29

**Authors:** Shanthi Mendis, Ian Graham, Jagat Narula

**Affiliations:** 1 Geneva Learning Foundation, Geneva, Switzerland; 2 Department of Cardiovascular Medicine, Trinity College, Dublin, Ireland; 3 Division of Cardiology, Mount Sinai Morningside, USA

**Keywords:** health policy, Prevention, cardiovascular disease, tobacco use, physical inactivity, unhealthy diet: total cardiovascular risk, harmful use of alcohol, sustainable development goal, low- and -middle- income countries, sustainability, scalability

## Abstract

Only 14 countries are on track to attain the Sustainable Development Goal (SDG) target of reducing premature mortality from Noncommunicable Diseases (NCDs) by one-third by 2030. This target cannot be reached without reducing the burden of cardiovascular diseases (CVDs) which is the major contributor to premature mortality from NCDs. Sustainable and scalable national responses to address both CVDs and their risk factors are urgently needed. Although smoking rates have decreased globally, consumption of alcohol and physical inactivity are on the rise. No country is on course to achieve the target to halt the rise in obesity or to reduce salt intake: targets critical for reducing the diabetes related cardiovascular burden and for hypertension control. Although very cost-effective scalable interventions are available, they are underutilized. Unless pathways selected to tackle CVDs prioritize prevention, primary health care and universal health coverage, countries will fall further behind in the attainment of the SDG target.

## Introduction

Cardiovascular disease (CVD) remains the major cause of morbidity and mortality world-wide. Although cost-effective interventions are available for addressing the CVD burden the global progress in their implementation has been too slow. Population and high-risk strategies are complementary and both are necessary to contain CVD and cardiovascular risk factors. Reliance on medications and tertiary care interventions, which is expensive, may be important for the relatively small number of very high-risk persons, but cannot solve the global problem.

This paper reviews the size of the problem, the disparities between high-income countries (HIC) and low-and-middle income countries (LMIC), current risk factor levels, affordable preventive and management strategies, progress in the implementation of cost-effective interventions and calls attention to the need for prioritizing scalable and sustainable frameworks in addressing the CVD burden, particularly in LMIC.

## Cardiovascular diseases: deaths, death rates and premature mortality

**Figure 1:**
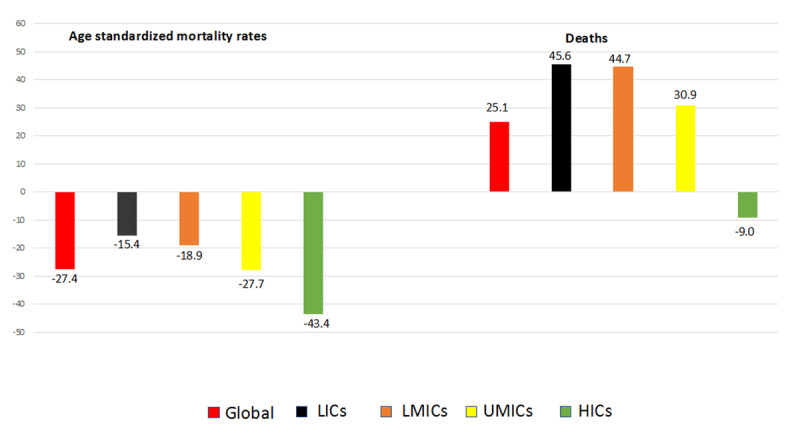
Changes in age-standardized mortality rate and deaths for cardiovascular diseases, by World Bank income group, 2000–2019 [2]. Source: WHO World Health Statistics 2021 [2].

The leading Noncommunicable Disease (NCD) throughout the last two decades has been CVD. In 2008, there were 17 million deaths due to CVD (48% of NCD deaths) and over 80% of cardiovascular deaths, occurred in LMIC [1]. Despite the reduction in age-standardised CVD mortality noted below, the total global CVD deaths have increased over the last two decades. There were 17.9 million CVD deaths in 2019, a 25.1% increase compared to 2000 (Figure 1), due to population growth, population ageing and fatal cardiovascular events in people with diabetes.

From 2000 to 2019, globally, there has been a 27.4 % decline in the age-standardized CVD mortality rate for all ages [2] (Figure 1). The decline has been greater in HIC (43.4%) compared to LMIC (27.7% in Upper- Middle -Income countries (UMIC), 18.9% in lower- middle- income countries and 15.4% Low- Income Countries (LIC) [3].

Ischaemic heart disease is responsible for the highest risk of premature death in more than half of all countries for women, and more than three-quarters for men [4]. Global premature NCD mortality – as measured by the probability of dying from one of the four major NCDs (CVD, cancer, diabetes and chronic respiratory diseases) between the ages 30 and 70 years (SDG indicator 3.4.1) – dropped from 22.9% in 2000 to 17.8% in 2019. The decline was approximately 30% in HICs and UMIC, but was only 13% to 16% in LIC and lower-middle-income countries [2].

Downward trends in premature NCD mortality are driven mainly by declines in tobacco use, population-level improvements in some biological risk factors such as blood pressure and advances in treatment of CVD [5,6,7].

## Cardiovascular risk factors: current status

Tobacco use, harmful use of alcohol, physical inactivity and unhealthy diet are the main behavioural risk factors that drive the global CVD epidemic. Biological factors include hypertension, dyslipidaemias and diabetes mellitus.

Smoking rates have decreased from a global average of 22.7% in 2007 to 17.5% in 2019, showing a relative reduction of 23% over 12 years. The relative reduction during this period was 20% in HIC, 19% in LIC and only 12% in middle income countries [8].

The risk of both ischaemic heart disease and ischaemic stroke is increased by irregular heavy drinking and consumption of high volumes of alcohol [9,10]. Alcohol consumption also increases the risk of hypertensive heart disease, cardiomyopathy, arrhythmias and non-ischemic strokes [9,10,11,12]. Globally in 2016, alcohol caused an estimated CVD burden of 593,000 deaths (3.3% of all CVD deaths) and 13 million Disability Adjusted Life Years (DALYs): 3.2% of all CVD DALYs [8]. Since 2000, the percentage of drinkers in the world has decreased from 47.6% to 43.0%. However, the total alcohol per capita consumption has increased globally between 2000 (5.7 litres of pure ethanol) to 6.4 litres in 2016 [8]. This may indicate that drinkers, although fewer in numbers, have increased their per capita consumption in most parts of the world.

Despite the strong evidence on the adverse health impact of physical inactivity and sedentary behaviours, worldwide, 1 in 4 adults, and 3 in 4 adolescents (aged 11–17 years), do not currently meet the global recommendations for physical activity, set by the World Health Organization [13].

An analysis of food consumption across 187 countries has shown that, between 1990 and 2010 diets based on unhealthy items have worsened globally [14]. The unhealthy items included, unprocessed red meat, processed meat, saturated fat, trans-fat, sodium, cholesterol and sugar sweetened beverages. According to the Global Nutrition Report in 2021 [15], no country is on course to achieve the target on reducing salt intake or to halt the rise in adult obesity. Further, no region in the world met the recommendations for healthy diets. Globally, percentage deviation below recommended minimum intake for healthy items was wholegrain 61%, fruits 60% and vegetables 40%. Percentage deviation above maximum recommended intake was 377% for red and processed meat. LIC continue to have the lowest intakes of key health-promoting foods such as fruits and vegetables, while HIC have the highest intakes of unhealthy foods, including red meat, processed meat and dairy products.

As a result of inadequate physical activity and unhealthy diet, in 2019, obesity accounted for approximately 5 million deaths from NCDs, which corresponded to 12% of all NCD deaths. Reaching the global target of zero growth in obesity and diabetes is critical to addressing the burden of CVD and achieving the SDG target 3.4 by 2030 of reducing by one-third premature mortality from NCDs by 2030.

## Cardiovascular interventions scalable and affordable to all countries

There are three essential, complementary and synergistic strategies for addressing the burden of CVD. They are, 1) Implementation of healthy public policies that reduce exposure of the population to behavioural and environmental risk factors (population-wide primary prevention) 2) Early detection and management of behavioural and biological risk factors using integrated interventions in primary health care (primary prevention at individual level) 3) Prevention of recurrent heart attacks and strokes in individuals with established CVD (secondary prevention).

Although a wide range of CVD interventions are available for implementing these three strategies, only 14 of them are cost effective enough to be affordable, scalable and sustainable in LMIC (Table 1). Since 2011, the World Health Organization has recommended that these interventions (best buys) be prioritized particularly in LMICs, given their relatively low levels of per capita health expenditure and the weak capacity of health systems [16]. Per capita spending on health is US$ 40 (38–43) in LIC, US$ 81 (74–89) in LMIC, US$ 491 (461–524) in UMIC and US$ 5252 (5184–5319) in HIC [17]. Although expenditure per head has roughly doubled between 2000 and 2016, from US$ 130 to US$ 270 in UMIC and from US$ 30 to US$ 58 in LMIC [18], LIC are still unable to fund a basic package of health services at current levels of health spending [19]. Further, WHO estimates a projected shortfall of 18 million health workers by 2030 mostly, in LMIC [20]. In this context, it is imperative that LMIC invest in high impact, scalable CVD interventions. Single risk factor programs that target all grades of hypertension, hyperlipidemia and diabetes with drugs ignoring nonpharmacological policy interventions and total cardiovascular risk assessment are neither affordable nor sustainable for LMIC [21].

**Table 1:** WHO best buys – very cost effective and scalable NCD interventions [5].


RISK FACTOR/DISEASE TO BE ADDRESSED	INTERVENTIONS	DETAILED DESCRIPTION

Reduce tobacco use	1. Taxation 2. Packaging 3. Advertising, promotion and sponsorship 4. Smoke free public policies 5. Health education	Increase excise taxes and prices on tobacco products. Implement plain/standardized packaging and/or large graphic health warnings on all tobacco packages. Enact and enforce comprehensive bans on tobacco advertising, promotion and sponsorship. Eliminate exposure to second-hand tobacco smoke in all indoor workplaces, public places, and public transport. Implement effective mass media campaigns that educate the public about the harms of smoking/tobacco use and second-hand smoke.

Reduce harmful use of alcohol	6. Taxation 7. Advertising 8. Availability	Increase excise taxes on alcoholic beverages. Enact and enforce bans or comprehensive restrictions on exposure to alcohol advertising (across multiple types of media). Enact and enforce restrictions on the physical availability of retailed alcohol (via reduced hours of sale).

Reduce unhealthy diet	9. Reformulate food 10. Supportive environment 11. Health education 12. Packaging	Reduce salt intake through the reformulation of food products to contain less salt and the setting of target levels for the amount of salt in foods and meals. -Reduce salt intake through the establishment of a supportive environment in public institutions such as hospitals, schools, workplaces and nursing homes, to enable lower sodium options to be provided. Reduce salt intake through a behaviour change communication and mass media campaign. Reduce salt intake through the implementation of front-of-pack labelling.

Reduce physical inactivity	13. Health education	Implement community-wide public education and awareness campaigns for physical activity which includes a mass media campaign combined with other community- based education, motivational and environmental programmes aimed at supporting behavioural change of physical activity levels.

Manage diabetes and cardiovascular disease including hypertension	14. Drug therapy and counselling	Drug therapy (including glycaemic control for diabetes mellitus and control of hypertension using a total risk approach) and counselling to individuals who have had a heart attack or stroke and to persons with high risk of a fatal and non-fatal cardiovascular event in the next 10 years.

Cervical cancer	15. Vaccination 16. Screening	Vaccination against human papillomavirus (2 doses) of 9–13 year old girls. Prevention of cervical cancer by screening women aged 30–49.


## Progress in implementation of the planned actions of the Global NCD Action Plan

The seventy-second World Health Assembly extended the period of the Global Action Plan for prevention and control of NCDs including CVD (NCD-GAP) to 2030, ensuring alignment with the 2030 agenda for sustainable development [22]. The NCD-GAP has six objectives- raise priority for NCDs; reduce exposure to risk factors; strengthen country capacity; Orient health systems; promote research and monitor trends and determinants. It is the principal guidance framework for national NCD plans and is underpinned by nine global targets [23] (Table 2). Targets are for reducing premature mortality, tobacco and alcohol use, salt intake, obesity, diabetes, and hypertension. Global targets 8 and 9 focus country action on individual prevention through early detection and treatment including through better access to medicines and primary health care. The target on prevention of heart attack and stroke, has been recently updated to reflect the revised CVD risk prediction charts [2]. A study based on modelling suggests that achieving the risk factor targets can contribute substantially towards meeting the premature NCD mortality target at the global and regional levels [25].

**Table 2:** Updated nine voluntary global NCD targets to be attained by 2030 [5].


**1.**	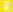	One third relative reduction in the overall mortality from CVD, cancer, diabetes or CRD.

**2.**	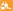	At least 20% relative reduction in the harmful use of alcohol.

**3.**	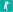	A 15% relative reduction in prevalence of insufficient physical activity.

**4.**	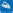	A 30% relative reduction in mean population intake of salt/sodium.

**5.**	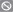	A 30% relative reduction in prevalence of current tobacco use.

**6.**	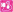	A 25% relative reduction in the prevalence of raised blood pressure or contain the prevalence of raised blood pressure.

**7.**	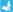	Halt the rise in diabetes and obesity.

**8.**	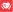	At least 50% of eligible people (age 40 years and older with a 10-year cardiovascular risk ≥20%) including those with CVD to receive drug therapy and counselling (including glycaemic control) to prevent heart attacks and strokes.

**9.**	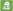	An 80% availability of the affordable basic technologies and essential medicines, including generics, required to treat major noncommunicable diseases in both public and private facilities.


Since 2013, the NCD-GAP, and the actions that flowed from it, such as the United Nations high-level meetings focused on NCDs, the establishment of the United Nations Inter-Agency Task Force on the Prevention and Control of NCDs and the establishment of a global coordination mechanism on the prevention and control of NCDs have helped to raise the international profile of NCDs and national-level responses. However, the raised profile given to NCD internationally since 2013 has not yet translated into increased international funding. In 2018, NCDs received only a small fraction (2%) of development assistance for health despite representing almost two thirds of the global disease burden [26]. This underscores the need for LMIC to focus attention on increasing domestic investment and to give top priority to affordable CVD interventions. Based on available data, overall, domestic spending on NCDs account for an average of US$ 23 per person per year in LIC US$ 214 in LMIC and US$ 527 in UMI [27].

Periodically over the last two decades, WHO has implemented a country capacity survey on NCDs as a means of assessing national-level responses to the NCD burden [28]. Country profiles, are published highlighting the latest data on NCD in each WHO Member State which allow them to track their progress towards achieving the nine global targets [29,30,31]. As shown in Tables 3 and 4, since 2013, the number of countries that have developed a policy, strategy or action plan to address NCD and CVD and set targets has increased. Modest progress has been made only in tobacco control. The robust global framework in place for tobacco (WHO Framework Convention on Tobacco Control) which limits industry interference may have contributed to more effective policy implementation. On the other hand, there has been little progress in policies to reduce harmful use of alcohol, physical inactivity and salt consumption. Some progress has been made on the percentage of countries able to provide drug therapy, including glycaemic control, hypertension control and counselling for eligible persons at high risk to prevent heart attacks and strokes. The percentage rose from 14% in 2013 to 36% in 2021. Several key areas which require accelerated action have been identified through country capacity surveys. These include broader adoption of fiscal policies, such as taxation on sugar-sweetened beverages and unhealthy foods, establishment of multisectoral mechanisms for policy coherence, implementation of health system best buys, and implementation research. The most recent WHO Progress Monitor highlights serious gaps in monitoring and surveillance. While no country in the WHO African Region has a system for periodic surveillance of NCD risk factors, no LIC has established a functioning system for generating reliable cause specific NCD mortality data on a routine basis [32].

**Table 3:** Percentage of countries implementing NCD-Global Action Plan, 2013 compared with 2021 based on Action Plan (AP) indicators (disaggregated data for 194 countries) [29,30,31,32,33].


INDICATOR	2013	2021

AP1: National action plan	24%	55%

AP2: NCD unit	51%	74%

AP3a: Policy on harmful use of alcohol	48%	69%

AP3b: Policy on physical activity	52%	72%

AP3c: Tobacco policy	63%	80%

AP3d: Policy on healthy diet	55%	84%%

AP4: Clinical guidelines	49%	58%

AP5: NCD research policy	n/a	28%

AP6: NCD surveillance system	23%	28%

APx: National coordination mechanism	n/a	46%


**Table 4:** Percentage of countries in which commitment fulfilment progress (COM) indicators are fully achieved; 2015 compared with 2021 [29,30,31,32,33].


INDICATOR	2015	2021

COM1: National NCD targets	30%	56%

COM2: Mortality data	36%	42%

COM3: Risk factor surveys	28%	19%

COM4: National action plan	33%	55%

COM5a: Tobacco tax	2%	20%

COM5b: Smoke-free places	25%	34%

COM5c: Graphic warnings	22%	53%

COM5d: Tobacco advertising bans	15%	29%

COM5e: Tobacco mass media	n/a	23%

COM6a: Alcohol sales restrictions	15%	16%

COM6b: Alcohol advertising ban	20%	27%

COM6c: Alcohol tax	22%	24%

COM7a: Salt policies	32%	17%

COM7b: Fat policies	21%	28%

COM7c: Child food marketing	22%	38%

COM7d: Breast milk code	37%	13%

COM8: Physical activity mass media	61%	42%

COM9: Clinical guidelines	26%	58%

COM10: Drug therapy and counselling	14%	36%


A midpoint evaluation of NCD GAP has been conducted in 2021. Results indicate that the effect of setting NCD policies, strategies and targets has been short-lived particularly in LMIC due to lack of long-term funding for their implementation [33]. Countries with a national NCD coordination mechanism are statistically more likely to have reduced the affordability of tobacco by increasing excise taxes and prices than countries without such a mechanism. Further, there is a statistically significant positive association between performance on many progress indicators and country income group. This was true for all Action Plan indicators expect one and more than half of the commitment fulfillment indicators. These findings also underscore the need for paying attention to affordability and sustainability of national NCD and CVD responses in LMIC.

The COVID-19 pandemic has had a significant negative impact on the progress of national NCD responses worldwide. It has reduced the already limited human, financial and health system resources and increased CVD morbidity and mortality due to increased vulnerability of people with established CVD to COVID-19 [34]. Other global crises such as the war in Ukraine, increasing food insecurity, political and economic instability and the growing impact of climate change thwart any hope of major increases in international funding for CVD prevention and control, in the foreseeable future.

## Realistic pathways to prevent and control the CVD burden to attain the SDG target 3.4

This year, the 75^th^ World Health Assembly adopted several resolutions and action plans to accelerate the global progress needed for addressing NCD including CVD. These include 1) a roadmap to guide countries to reorient and accelerate their domestic action plans with a view to placing themselves on a sustainable path to meet the nine global NCD targets and SDG target 3.4 [35]; 2) an acceleration plan for prevention and management of obesity [36] and 3) an action plan to effectively implement the global strategy to reduce the harmful use of alcohol as a public health priority [37].

The NCD countdown 2030 collaboration has identified tobacco and alcohol control and primary and secondary cardiovascular disease prevention in high-risk individuals and hypertension and diabetes treatment as effective pathways to address NCDs [4]. Tobacco and alcohol control interventions need to be combined with community-wide public education and awareness campaigns to promote physical activity and programmes aimed at supporting behavioural change. These population based preventive interventions are among the best-buys and are affordable to all countries.

Health system interventions need to be implemented in such a way to target limited financial and workforce resources at individuals most vulnerable to heart attacks, strokes, heart failure and kidney failure [38]. This is critical particularly for resource constrained settings and LMIC. It can be done effectively through a total cardiovascular risk approach which bundles a set of risk factors – hypertension, diabetes, obesity, tobacco use, serum cholesterol – as entry points [4,39]. Tools and protocols available for implementing this intervention in primary health care also identify individuals with previous heart attacks and strokes, to ensure continuation of drug treatment (secondary prevention). In addition, simplified protocols enable drug treatment to be continued for individuals with diabetes, hypertension and hypercholesterolemia, as appropriate [24,40]. Providing access to a core set of essential medicines (aspirin, statin, angiotensin converting enzyme inhibitor, beta blocker, calcium channel blocker, metformin and insulin), for implementing these evidence based protocols at all levels of care is one of the nine global NCD targets (Table 2). Applying the total risk approach to individuals with diabetes is essential to minimize cardiovascular risk and prevent cardiovascular and renal complications.

Affordable and sustainable approaches are of primary importance in addressing hypertension which is a lifelong condition. Interventions to reduce salt consumption is a best buy for addressing hypertension and need to be implemented in all countries. The prevalence of hypertension (defined as equal to or above 140/90 mm Hg) is high in nearly all populations and reaches 50% or higher in those above the age of 60 years. It is important to avoid misdiagnosis and over treatment. Diagnosis needs to be confirmed using repeated measurements. Individuals with BP ≥ 160/100 mmHg, once confirmed, usually require lifelong drug therapy in addition to nonpharmacological interventions. Those with a BP ≥ 140/90mmHg and less than 160/100 mmHg in the absence of other cardiovascular risk factors could be offered a short-term trial of nonpharmacological measures at the first visit [5,40]. This helps to confirm the diagnosis of hypertension before commencing lifelong drug therapy, if necessary, at the 2^nd^ visit. Further, the blood pressure lowering effect of nonpharmacological interventions enable reduction of the dose of antihypertensive drugs, causing no harm to the patient. As hypertension has a high prevalence, these measures are critical for improving the efficiency of utilizing health care budgets in LMIC. In addition, to ensure sustainability, the total cardiovascular risk assessment and treatment need to be integrated into basic benefit packages in universal health coverage initiatives.

## Conclusion

Ischaemic heart disease and stroke are the biggest contributors to premature NCD mortality worldwide. Accelerating action to address the CVD burden is imperative in order to attain the global NCD targets and the SDG target of reducing premature mortality by one-third by 2030. The impact of CVD interventions, ensue relatively quickly. Interventions to address CVD which are affordable and scalable even to LMICs with limited health system resources are already available. They need to be prioritized in primary health care and Universal Health Coverage initiatives for sustainable implementation. If pathways taken to accelerate national NCD responses do not address CVD while giving priority to cost-effective CVD interventions implementable with limited resources, LMIC will fall further behind in the race to attain the SDG target 3.4 by 2030.
